# Dietary Pattern Associated with the Risk of Hyperuricemia in Chinese Elderly: Result from China Nutrition and Health Surveillance 2015–2017

**DOI:** 10.3390/nu14040844

**Published:** 2022-02-17

**Authors:** Yuxiang Yang, Wei Piao, Kun Huang, Hongyun Fang, Lahong Ju, Liyun Zhao, Dongmei Yu, Yanan Ma

**Affiliations:** 1 NHC Key Laboratory of Trace Element Nutrition, National Institute for Nutrition and Health, Chinese Center for Disease Control and Prevention, Beijing 100050, China; yxyang_ninhccdc@126.com (Y.Y.); piaowei@ninh.chinacdc.cn (W.P.); 15550807252@163.com (K.H.); fanghy@ninh.chinacdc.cn (H.F.); julh@ninh.chinacdc.cn (L.J.); zhaoly@ninh.chinacdc.cn (L.Z.); 2 Department of Biostatistics and Epidemiology, School of Public Health, China Medical University, Shenyang 110122, China; ynma@cmu.edu.cn

**Keywords:** dietary pattern, hyperuricemia, elderly, surveillance, factor analysis

## Abstract

Our current study aimed to estimate the relationship between dietary patterns and hyperuricemia among the Chinese elderly over 60 years old. All the data were obtained from China Nutrition and Health Surveillance during 2015–2017. A total of 18,691 participants who completed the whole survey were included in our statistical analysis. The definition of hyperuricemia was 420 μmmol/L (7 mg/dL) for male and 360 μmmol/L (6 mg/dL) for female. Exploratory factor analysis was applied to explore posterior dietary patterns in our samples, and five dietary patterns were recognized, namely “Typical Chinese”, “Modern Chinese”, “Western”, “Animal products and alcohol”, and “Tuber and fermented vegetables”. After multiple adjusted logistic regression, participants in the highest quartile of “typical Chinese” (Q4 vs. Q1, OR = 0.32, 95% CI: 0.28–0.37, *p*-trend < 0.0001), “modern Chinese” (Q4 vs. Q1, OR = 0.81, 95% CI: 0.71–0.93, *p*-trend = 0.0021) and “tuber and fermented vegetables” (Q4 vs. Q1, OR = 0.78, 95% CI: 0.69–0.88, *p*-trend < 0.0001) showed a lower risk of hyperuricemia, while animal products and alcohol was positively associated with hyperuricemia (Q4 vs. Q1, OR = 1.49, 95% CI: 1.31–1.7, *p*-trend < 0.0001). We also found that participants who mainly ate a modern Chinese diet tended to meet the RNI/AI of nutrients we discuss in this paper, which may supply some information for hyperuricemia prevention and management by dietary methods.

## 1. Introduction

Uric acid (UA) is the terminal metabolite of purine, as one of the antioxidant compounds in the human body that has a function in antioxidation, e.g., inhibiting DNA from getting damaged [1]. However, recent studies mainly focus on its harmful effect considering the background of the spiking incidence of hyperuricemia (HUA) during the last few decades in developing and developed countries, which has become an emerging public health problem. The causation of HUA is the elevated concentration of UA in plasma accompanied by urate overproduction or (and) impairment of the urate-excreting function of the kidney and gastrointestinal tract [2]. It is one of the risk factors leading to gout. It might play an essential role in the pathogenesis of several noncommunicable diseases (NCDs) such as diabetes, hypertension, cardiovascular disease, and chronic renal disease [3].

Since many NCDs are age-related, aging people have become one of the most susceptible groups to those NCDs [4]. It is estimated that there will be over 350 million elderly people in China [4]. However, with the growing life expectancy, the burden of NCDs will also increase. In China, it is reported that the prevalence of HUA in the elderly is at a high level (60–69 years: 25.5%, 70+ years: 18.2%) [5], and those with HUA are more likely to be affected by adverse health outcomes [6]. With the rapid pace of population aging, there is an urgent need to lower their risk of NCDs by adopting multiple measures for the well-being of aging people, which would further benefit global sustainable development and healthy aging progress [7].

It is declared that diet, as a determinant of HUA [8], could exert either positive or negative effects in HUA prevention [6]. Current studies show that low-purine food such as vegetables, fruits, soybean products, dairy, and related products can lower the level of serum UA. In contrast, foods that contain high purine levels, such as meats, seafood, and alcoholic beverages, increase the risk of HUA [9]. However, it has been reported that a single food or nutrient may not reflect the whole condition of one’s diet. More researchers have turned their focus to evaluating dietary patterns (DPs) instead of a part of the diet in nutritional epidemiological studies [10]. Previous studies about DPs in this field, which were mainly based on prior methods, reported that both the DASH diet and Mediterranean diet can alleviate the hazard of HUA [8,11,12,13]. Still, these two DPs are fairly different from that of the Chinese. Posteriori methods represented by factor analysis (principal component analysis) or cluster analysis could find the DPs in the studied sample more accurately and authentically [10]. Plus, few studies evaluated the association between DPs and HUA among the aging Chinese. Thus, we applied the data from China Nutrition and Health Surveillance 2015–2017 to explore whether some DPs in Chinese people aged over 60 years old were linked with HUA.

## 2. Materials and Methods

### 2.1. Participants

The cross-sectional data were from the China Nutrition and Health Surveillance (2015–2017) (CNHS 2015–2017). The survey was conducted among adults 18 years and older in 2015. This study enrolled its participants in 2015. Sampling design was based on a stratified, multistage, and random sampling method to extract the representative samples from 31 provinces/municipalities/autonomous in mainland China in 2015. Given the consideration to ensure the distribution balance of stratification factors and working conditions, we finally selected 302 monitoring locations to conduct the whole survey. Further information is stated elsewhere [14]. The including criteria were as follows: (1) participants aged 60 years old or older; (2) completed all the parts of the components including basic information interview, dietary survey, body measurement, and laboratory test; and (3) individuals with average energy intake, which is 800–4800 Kcal for males and 500–4000 Kcal for females after calculated by Food Frequency Questionnaire [4]. We finally included 18,765 participants in current study. All the participants signed the informed consent at the beginning of the survey, which was supported by the Ethics Committee of the Chinese Center for Disease Control and Prevention (approval number: 201519-B).

### 2.2. Basic Information Interview

Well-trained health investigators collected all the household and individual basic information (urban or rural, income, educational level, marital status, smoking and alcohol drinking, physical activities, etc.). The information of interviewees in the questionnaire was collected in a face-to-face manner by investigators.

### 2.3. Dietary Assessment

Dietary information was assessed by a validated food frequency questionnaire (FFQ) in CNHS 2015–2017, which collected their dietary habits during the past 12 months. The 64 food items on the questionnaire varied from staple foods, soybeans and its products, vegetables, fruits, dairy and its products, meats, aquatic products, eggs, and other kinds. The beverages were categorized into two main types, soft drinks and alcoholic beverages. The daily eating weight was calculated based on frequency (daily, weekly, monthly, or yearly), and the edible weight was defined as weight consumed in a single day. Foods that participants had never eaten before were recorded as zero. As for edible oil and condiments, we recorded the consumption of their whole family in the last month and the number of family members who usually ate at home at each meal (breakfast, lunch, and dinner), and then calculated their average daily intake of edible oil and condiments. Then, we summed up each person’s daily energy and nutrient intake by their daily food, edible oil, and condiment intake based on the China Food Composition Table (2009) and China Food Composition (2018) [15,16]. The parts derived from nutrient supplements were not included.

### 2.4. Clinical Examination

To reduce the bias of measurement, each item in this part was measured using the same type and brand of machines. For height, we used the TZG height meter. Participants’ weight was measured on an empty stomach in the morning using an electronic weight scale (TANITA HD-390). The measurement of blood pressure was taken using an electronic sphygmomanometer (OMRON HBP1300). The accuracy of those instruments was 0.1 cm, 0.1 kg, and 1 mmHg, respectively.

### 2.5. Laboratory Test

A total of 8 mL of overnight fasting blood was drawn at one time to measure fasting glucose, total cholesterol, triglyceride, LDL-C, HDL-C, blood uric acid, and glycohemoglobin. All the above measurement were taken by professionals in the laboratory with strict quality control.

### 2.6. Definition of HUA and Other NCDs

The definition of health outcome was based on the concentration of UA in plasma. HUA was defined according to the clinical diagnostic criteria. The cut-off serum UA value for men was 420 μmmol/L (7 mg/dL) and 360 μmmol/L (6 mg/dL) for women [2]. In addition to those who reported they had been diagnosed by a township health center or community health service center or higher-level medical institutions, we also considered those participants whose fasting plasma glucose level ≥ 7.0 mmol/L or glycohemoglobin level ≥ 6.5% as diabetic [2], those whose mean systolic blood pressure ≥ 140 mmHg and (or) mean diastolic blood pressure ≥ 90 mmHg and (or) received antihypertensive medicine within two weeks as hypertensive [17], and those with total cholesterol ≥ 6.2 mmol/L or triglyceride ≥ 2.26 mmol/L or LDL ≥ 4.14 mmol/L or HDL < 1.04 mmol/L as hyperlipidemic [18]. Regarding coronary heart disease, participants with any kind of this disease or those taking related medical treatment were considered as sufferers. Patients with either ischemic stroke (e.g., cerebral thrombosis, infarction, embolism, etc.) or hemorrhagic stroke (e.g., cerebral or subarachnoid hemorrhage, etc.) were counted as apoplectic. All the above diseases were used to calculate the number of NCDs among those surveyed.

### 2.7. Covariates

The variety of variables that were used for multiple adjustments in the logistic regression analysis were defined as follows. (1) Living area was separated into urban and rural. (2) Education status was divided into primary school or below, middle school, and high school or higher. (3) Income levels were divided into low (<10,000 RMB/year), medium (10,000–25,000 RMB/year), and high (>25,000 RMB/year) based on household income per capita. (4) Marital status was separated into living with spouse and other status. (5) Body mass index (BMI) was categorized as underweight (BMI < 18.5), normal (18.5 ≤ BMI < 24), overweight (24 ≤ BMI < 28), or obese (BMI ≥ 28) according to China Working Group on Obesity [17]. (6) Current smoker was categorized as yes or no, regardless of how often they smoked. (7) Alcohol drinking status was categorized as yes or no in the last 30 days. (8) Sedentary behavior was categorized as less than 2 h/day, 2–3 h/day, or over 4 h/day. (9) Sleep status was categorized as less than 6 h/day, 6–9 h/day, or over 10 h/day. (10) Physical activity status was decided based on weekly total metabolic equivalent (MET) and total weekly duration of different exercise levels: low (MET < 600), moderate (600 ≤ MET ≤ 3000), or high (MET > 3000) [19,20]. (11) Number of NCDs was further divided into 2 categories: less than 1 kind and over 2 kinds.

### 2.8. Dietary Pattern

A total of 64 food items in FFQ were categorized into 27 food groups and calculated for daily consumption weight using factor analysis with varimax rotation to explore the latent Dietary Pattern (DP) in our samples. Each DP was named by the characteristics of food variables whose absolute factor loading was over 0.2 [21]. Those food variables with higher factor loadings within DPs indicated a higher consumption. Conversely, factor loadings with negative value indicated less. We also calculated DP scores of each DP for every participant. A higher score indicated one’s diet was closer to the corresponding DP. Then, subjects were divided into four groups according to quartile of each DP score. Every participant’s representative DP was considered the DP whose score was highest compared to the others.

### 2.9. Nutrient Intake Assessment

Nutrient intake was assessed by 7 kinds of nutrients such as calcium, magnesium, zinc, selenium, Vitamin B_1_/C/E, and dietary fiber. Except for calcium, the rest were reported to have a positive effect on the prevention of HUA [2,22,23,24,25,26,27]. However, calcium is an essential mineral for skeletal health and NCD prevention [28] so we also included it in our current study. The cut-off value was defined as the Recommended Nutrient Intake (RNI) or Adequate Intake (AI) of the above nutrients and dietary fiber based on the Chinese Dietary Guidelines (2016) for each gender and age group [29]. Then, we calculated and compared the proportion of participants with each DP who met the recommendation of several nutrients.

### 2.10. Statistical Analysis

SAS version 9.4 software (SAS Institute Inc, Cary, NC, USA) was used for all statistical data cleaning and analysis and R version 4.1.2 for plot drawing in this study. The continuous variables of the sample’s baseline characteristics were presented by the mean and standard deviation (normal distribution data) or median and IQR (abnormal distribution data), counts, and percentage for categorical variables. In order to keep national representativeness when describing the prevalence of HUA and related 95% confident interval, PROC SURVEYFREQ program in SAS was applied. The weight of the sample was accessed by data from China National Bureau Statistics in 2010. Factor analysis was conducted by PROC FACTOR in SAS. Using multiple logistic regression to examine the association between each dietary pattern and HUA, the results were given by odds ratio (OR) and 95% confidence interval (95% CI) compared with the reference quartile (the lowest one) in different models. Model 1 was a crude model without any adjusted covariables. Model 2 was adjusted for age and BMI. Model 3 was further adjusted for location, income, education, marital status, smoke, drinking, physical activities, sleeping time, and total energy intake. All the covariables were transferred into dummy variables before conducting the adjustive process. The comparison of continuous variables between different groups was completed by t-test or nonparametric statistical hypothesis test based on the normality of variables. The chi-square test checked the statistical difference of categorical variables among the groups. The definition of statistical significance was two-tailed and *p* < 0.05.

## 3. Results

### 3.1. Characteristics of Participants

Characteristics of 18,691 elderly people are displayed in Table 1. The number of females was slightly higher than males (50.07% vs. 49.93%). This difference was also observed when comparing BMIs between different gender groups. However, the median BMI value in males was at a normal level. In contrast, the median in females was overweight, which showed older men performed better than elderly women in BMI (23.68 vs. 24.25). Male participants were slightly older than females (66.89 vs. 66.11). The comparison of living location (urban or rural), education, marital, smoking and alcohol-drinking status, physical activity, static status, sleeping time, and the number of chronic disease between different genders also showed significant differences except for income level (*p*-value = 0.2021). As for clinical indicators, the median level in male participants tended to be slightly less than in females except for diastolic pressure and UA.

### 3.2. Weighted Prevalence of HUA and Its Distribution

**Figure 1:**
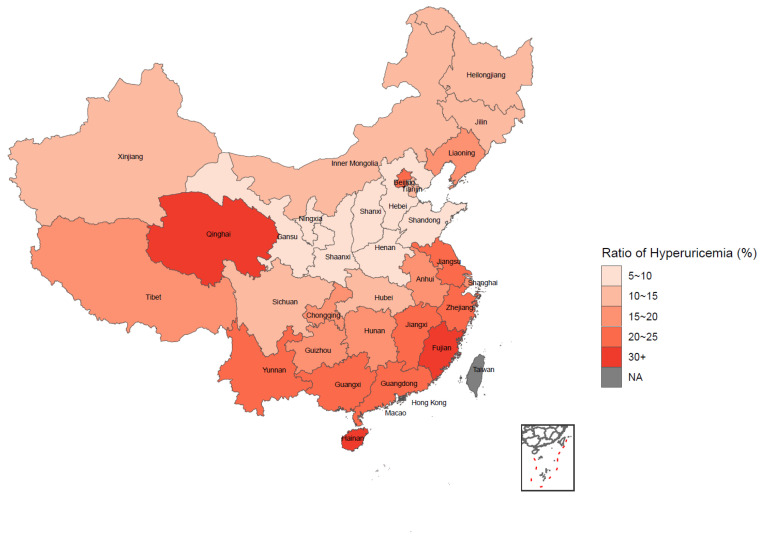
Distribution of hyperuricemia among Chinese elderly by weighted prevalence.

Table 2 shows the weighted prevalence of HUA by different categories, and the statistical inference was completed by the Rao–Scott chi-square test. The prevalence of HUA among all the participants was 15.73% in general. The prevalence of HUA was significantly different under different categories of age, BMI, education, income, alcohol drinking, sedentary behavior, and chronic disease condition. Generally, participants who were older and had a higher level of BMI, education, income and sedentary behavior, those with more NCDs, and alcohol drinkers tended to have a higher prevalence of HUA. However, no statistical significance was observed when comparing the prevalence among different gender, marital status, smoking, physical activity, and sleeping time groups among participants. As for the nationwide distribution in Figure 1, a trend could be observed that the weighted prevalence of HUA gradually increased from the northwest to southeast and from the inland to coastal areas in China. Further information on the weighted prevalence of HUA among different regions in China is available in Supplementary Table S1.

### 3.3. Dietary Patterns among Chinese Elderly

**Figure 2:**
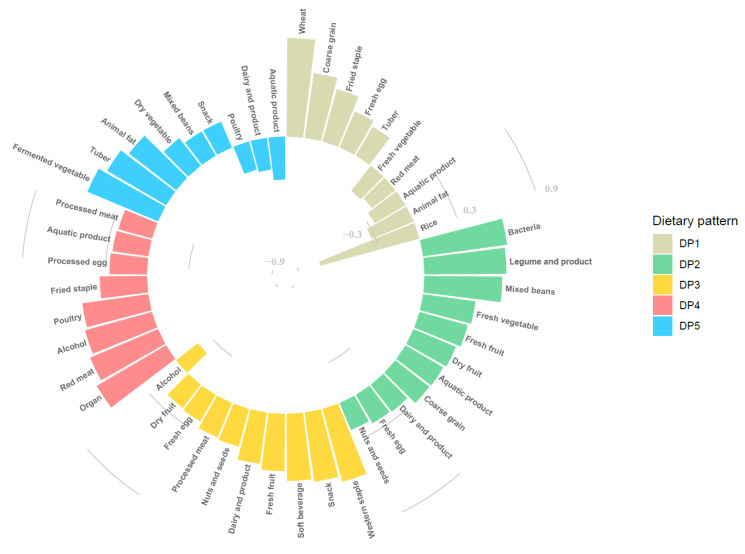
Factor loading of food items in each dietary pattern.

**Figure 3:**
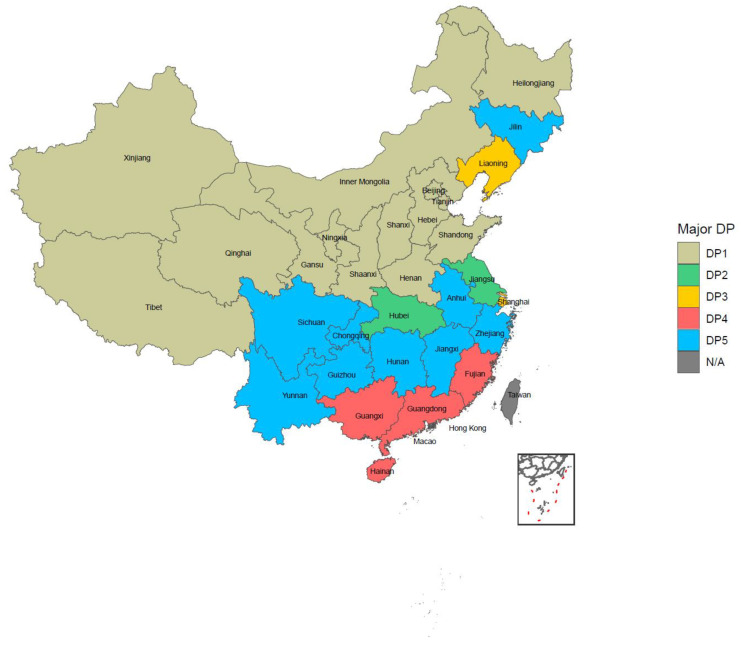
The distribution of dietary patterns among elderly on China mainland.

We finally extracted five dietary patterns by factor analysis, which totally explained the 30.37% variance of all 27 food variables. DP1 was named as the “Typical Chinese pattern”, DP2 as the “Modern Chinese pattern”, DP3 as the “Western pattern”, DP4 as the “Animal products and alcohol pattern”, and DP5 as the “Tuber and fermented vegetables pattern” based on the characteristics of factor loadings, which were 0.2 or higher in each dietary pattern, as shown in Figure 2. Further information on factor loadings is available in Supplementary Table S2. The distribution of DPs in China is shown in Figure 3. The DP with the highest proportion in each province/municipality/autonomous was considered as the representative DP in that area. All the typical patterns existed in the north of China. The modern Chinese pattern showed in both the Jiangsu and Hubei provinces. The Western pattern appeared in the Liaoning province and Shanghai city. The animal products and alcohol pattern and tuber and fermented vegetables pattern were mainly in southern China. The former was presented in the inland parts, while the latter was in the coastal regions. The proportion of each DP in detail is available in Supplementary Table S3.

### 3.4. Association between Dietary Patterns and Hyperuricemia

Results are shown in Table 3 to explain the association between dietary patterns and HUA by logistic regression. There were three DPs that had a negative association with HUA, namely the typical Chinese pattern (Q4 vs. Q1, OR = 0.32, 95% CI: 0.28–0.37, *p*-trend < 0.0001), modern Chinese pattern (Q4 vs. Q1, OR = 0.81, 95% CI: 0.71–0.93, *p*-trend = 0.0021) and tuber and fermented vegetables pattern (Q4 vs. Q1, OR = 0.78, 95% CI: 0.69–0.88, *p*-trend < 0.0001) in the multiple-adjusted model. Meanwhile, a positive association was observed when describing the relationship between the animal products and alcohol pattern and HUA. The risk of HUA in the highest quartile was nearly 50% higher than the lowest quartile (Q4 vs. Q1, OR = 1.49, 95% CI: 1.31–1.7, *p*-trend < 0.0001). For the Western pattern, no significant association was found except for a positive linkage with HUA (Q4 vs. Q1, OR = 1.21, 95% CI: 1.09–1.35, *p*-trend = 0.0009) in the crude model.

### 3.5. Proportion of Participants Who Reached RNI/AI under Each Dietary Pattern

**Figure 4:**
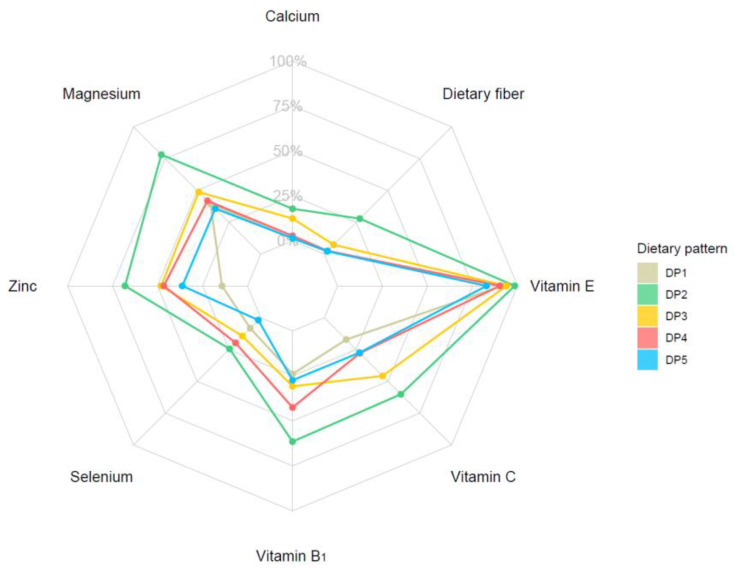
The proportion of participants with each DP who reached the RNI/AI standard of nutrients.

The evaluation of the proportion of participants who had less than five kinds of DPs who had met the RNI/AI of eight minerals/vitamins/dietary fiber is shown in Figure 4, and further information is available in Supplementary Table S4. For all the kinds of nutrients and dietary fiber we included in this article, the modern Chinese pattern achieved a significantly higher percentage. The poorest intake of calcium (1.29%), zinc (14.21%), Vitamin B_1_ (23.98%), Vitamin C (17.17%), and dietary fiber (2.12%) was in the typical Chinese pattern, while magnesium (35.7%), selenium (1.9%) and Vitamin E (82.77%) levels were lowest in the tuber and fermented vegetables pattern.

## 4. Discussion

Besides the genetic factor, considerable and intervenable factors relate to the prevention of HUA [30]. Among them, diet is considered as a critical determinant [2]. According to researchers, 2/3 of total uric acid in our body is produced in the endogenous pathway. Around 1/3 of uric acid in serum comes from dietary purine metabolism [31], a poor dietary quality which was represented by lower plant intake, and higher animal product and alcoholic beverage intake is linked to HUA. Since many people favor nonpharmacological options for disease prevention and treatment, even for those taking medicine, diet could still serve as an adjuvant therapy to improve their health condition [32].

Several dietary patterns have already been identified relating to HUA. However, few studies have explored the association between HUA and DPs among the Chinese elderly and executed the comparison of nutrient intake between several DPs. The current research examined five dietary patterns among 18,691 Chinese elderly samples: the typical Chinese pattern, modern Chinese pattern, Western pattern, animal products and alcohol pattern, and tuber and fermented vegetables pattern. A higher DP score in the typical, modern, and tuber and fermented vegetable patterns showed a protective effect against HUA. In contrast, animal products and alcohol patterns had a higher risk after adjusting multiple variables in the logistic model. However, our current study observed no significant linkage between the Western pattern and HUA.

Similar results were found in current studies about DPs and HUA among general Chinese adults. Zhang et al. reported in their results that the highest scoring group with the pattern labeled “animal products and fried food” had an increased risk of HUA when compared with the lowest tertile, and the “soybean products and fruit” pattern could decrease the risk. Additionally, there was no significant result for the Western pattern in their study [33]. Another cross-sectional study conducted among 45- to 59-year-old Chinese adults indicated the traditional Chinese pattern had a lower risk of HUA, conversely, greater risks were shown in the meat pattern, and there’s no statistically significant result was observed in the mixed food pattern [34]. Zhang et al. conducted a prospective cohort study in this field. Using data from the TCLSIH cohort, researchers identified three main DPs: the vegetable, sweet food, and animal food pattern. After establishing multiple-adjustment Cox proportional hazards regression, the vegetable pattern was the most protective one, while both sweet and animal food patterns could increase the incidence of HUA [10].

The three DPs that had a negative association with HUA were dominantly represented by low-purine foods, such as foods rich in carbohydrates (wheat, coarse, fried pasta, tuber), both fresh and dry vegetables and fruits, fermented vegetables, and dairy products and eggs, and less consumption of those rich in purine, including meat and related products, aquatic foods, and alcoholic beverages. Interestingly, the modern Chinese pattern showed higher factor loading (>0.6) in purine-rich foods such as bacteria (mushrooms and another edible fungus), legume products, and mixed beans. Our results precisely match the report that eating purine-rich vegetables couldn’t increase the risk of HUA [6,35]. Plus, these kinds of foods benefit both human health and intestinal probiotics in the human body, which could lead to further benefits for human health [36]. We concluded the tuber and fermented vegetable pattern had a protective effect on the risk of HUA at this time. The inner mechanisms may not simply be due to a lower consumption of purine-rich food and a higher consumption of fermented vegetables such as kimchi, which contains various probiotics such as *Lactobacillus* [37]. Evidence was found by Xiao et al. that *Lactobacillus* could alleviate HUA in rats, which indicates its potential therapeutic effect on chronic HUA patients [38]. We also found that the riskiest patterns, such as the animal products and alcohol pattern, mainly comprised purine-rich food, especially organs, meat, and poultry. Alcohol, as the variable with the second most significant factor loading in this pattern, had also been proven as a risk factor of HUA in several studies [9].

Like former studies, we found that the modern Chinese pattern is more similar to the DASH and Mediterranean DPs, which include a higher intake of vegetables, fruits, legumes and nuts, and dairy products and lower intake of sweetened beverages and meats [8,11,12,13]. In the current study, we found that compared with Q1, there was a 19% lower risk in Q4 with statistical significance. Similar results emerged in recent studies which discussed the relationship between DASH and the Mediterranean diet and HUA. A 30-day randomized control trial was conducted in the United States to examine the effect of the DASH diet on lowering serum UA among a total of 103 adults with pre- or stage 1 hypertension. The intervention group was given the food under the rules of the DASH diet, while the control group was provided with food common in the typical Western DP. Finally, researchers found after 30 days of intervention, the DASH diet had reduced serum UA with a higher effect among eight participants who had already had an HUA at the baseline level [8]. A prospective study involved 44,444 male participants from the Health Professionals Follow-up Study (HPFS), which showed that after the 26-year follow-up, compared with the lowest-DASH-score group, samples which had the highest compliance to the DASH diet had a lower risk of gout, suggesting the effect of DASH diet to lower the uric acid levels, and then to reduce the risk of gout. Simultaneously, a higher tendency to following a Western diet pattern, which was similar to the animal products and alcohol pattern in our study, mainly composed of meat, processed meat, and fried food, was positively associated with gout among the participants [12]. According to the results from the ATTICA study, which indicated that the MedDietScore (a measurement to assess compliance to the Mediterranean diet), as an independent factor, had a negative linkage with serum UA levels; participants at the highest quartile only had a 30% risk of HUA compared to the lowest quartile [11]. The above studies suggest the DASH and Mediterranean diet could play a potential role in HUA prevention and treatment.

Three protective DPs of HUA were separated in our study, and it was not difficult to find out the typical Chinese pattern had the most obvious effect on lowering the risk of HUA. Nevertheless, when we considered the intake level of nutrients, the story became different. When we analyzed the proportion of participants in each DP who met those nutrients’ RNI or AI standard, we found that those with the modern Chinese pattern performed better. However, those with the typical and tuber and fermented vegetable pattern performed worse at this stage, suggested that when applying dietary intervention to HUA prevention and treatment, one of the critical parts is evaluating whether the aim diet could supply an amount of nutrients equal to the daily needs of the target individual or groups.

To the best of our knowledge, the present study was the first to explore the relationship between DPs and HUA among the Chinese elderly population. The strengths of our study were as follows. First, we obtained the results by using nationwide data on the Chinese elderly population. Second, all the data were collected by well-trained professionals who performed quality control in every process, ensuring the reliability of the information. Third, we considered several covariables when setting the logistic regression model to obtain results that were as close to the reality as possible. However, there were still some limitations that should be noted. First, due to the inherent limitations of the cross-sectional study, it was difficult to draw the causal relationship between DPs and HUA. Our findings need to be verified in further prospective studies. Second, we applied the FFQ questionnaire to evaluate the dietary intake during the whole year, which may cause recall bias. However, it is the most frequently used method to obtain dietary information for DP exploration. Third, though we had controlled several covariables, we faced difficulties in controlling the remaining unmeasurable variables.

## 5. Conclusions

Among Chinese elderly people in the current study, the patterns of “Typical Chinese”, “Modern Chinese” and “Tuber and fermented vegetables” were positively associated with HUA. At the same time, “Animal products and alcohol” was a risk factor for HUA. Diet might serve as an adjuvant therapy to improve human health conditions. Hence, it indicates the new prospect of HUA prevention through effective dietary intervention and may have further benefits in preventing whole noncommunicable chronic diseases among elderly people and assist in achieving healthy aging.

## Figures and Tables

**Table 1:** General characteristics of elderly participants in CNHS 2015–2017 by gender group.

	Male	Female	Total
N (%)	9332 (49.93)	9359 (50.07)	18,691
Age (years) *	66.89 (63.19, 72.10)	66.11 (62.77, 71.18)	66.51 (62.97, 71.69)
BMI (kg/m^2^) *	23.68 (21.36, 26.11)	24.25 (21.91, 26.81)	23.96 (21.62, 26.46)
Urban or rural *			
Urban	4148 (44.45)	4365 (46.64)	8513 (45.55)
Rural	5184 (55.55)	4994 (53.36)	10,178 (54.45)
Education*			
Primary school or below	5462 (58.53)	7171 (76.62)	12,633 (67.59)
Middle school	2436 (26.1)	1417 (15.14)	3853 (20.61)
High school or higher	1434 (15.37)	771 (8.24)	2205 (11.8)
Income (CNY)			
Low	3819 (40.92)	3710 (39.64)	7529 (40.28)
Medium	3384 (36.26)	3465 (37.02)	6849 (36.64)
High	2129 (22.81)	2184 (23.34)	4313 (23.08)
Marital status *			
Living with spouse	8712 (93.36)	8004 (85.52)	16,716 (89.43)
Other status	620 (6.64)	1355 (14.48)	1975 (10.57)
Current smoker *			
No	5071 (54.34)	8959 (95.73)	14,030 (75.06)
Yes	4261 (45.66)	400 (4.27)	4661 (24.94)
Alcohol drinking *			
No	4684 (50.19)	8126 (86.83)	12,810 (68.54)
Yes	4648 (49.81)	1233 (13.17)	5881 (31.46)
Physical activity *			
Low	2358 (25.27)	2092 (22.35)	4450 (23.81)
Moderate	2502 (26.81)	2489 (26.59)	4991 (26.7)
High	4472 (47.92)	4778 (51.05)	9250 (49.49)
Sedentary behavior (h) *			
0~<2	1066 (11.42)	1340 (14.32)	2406 (12.87)
2~3	3399 (36.42)	3483 (37.22)	6882 (36.82)
≥4	4867 (52.15)	4536 (48.47)	9403 (50.31)
Sleeping time (h) *			
0~<6	912 (9.77)	1368 (14.62)	2280 (12.2)
6~9	7131 (76.41)	6904 (73.77)	14,035 (75.09)
≥10	1289 (13.81)	1087 (11.61)	2376 (12.71)
NCDs *			
Less than one disease	5629 (60.32)	5224 (55.82)	10,853 (58.07)
Over two diseases	3703 (39.68)	4135 (44.18)	7838 (41.93)
Glu (mmol/L) *	5.35 (4.92, 5.91)	5.39 (4.99, 5.98)	5.37 (4.96, 5.94)
Tc (mmol/L) *	4.69 (4.12, 5.31)	5.07 (4.47, 5.72)	4.87 (4.27, 5.53)
Tg (mmol/L) *	1.14 (0.8, 1.67)	1.37 (0.98, 1.97)	1.25 (0.88, 1.83)
LDL (mmol/L) *	2.89 (2.37, 3.44)	3.19 (2.64, 3.78)	3.03 (2.49, 3.61)
HDL (mmol/L) *	1.24 (1.03, 1.49)	1.29 (1.09, 1.52)	1.27 (1.06, 1.51)
HbA1c (%) *	5.1 (4.6, 5.5)	5.2 (4.7, 5.6)	5.1 (4.7, 5.5)
SBP (mmHg) *	142 (129.67, 157)	144.33 (130.67, 160.33)	143 (130, 158.67)
DBP (mmHg) *	80.67 (73.67, 88)	78 (71, 85.67)	79.33 (72, 87)
SUA (μmmol/L) *	333 (281, 392.6)	275.9 (233, 328.3)	303.4 (252.3, 364.3)

Values of polytomous variables may not sum to 100% because of rounding. Abbreviation: BMI— body mass index; Glu—fasting blood glucose; Tc—total cholesterol; Tg—triglyceride; LDL—low density lipoprotein; HDL—high density lipoprotein; HbA1c—glycosylated hemoglobin; SBP—systolic blood pressure; DBP—diastolic blood pressure; UA—serum uric acid. * Indicated *p*-value < 0.05.

**Table 2:** Weighted prevalence of hyperuricemia in Chinese elderly in CNHS 2015–2017.

	Prevalence %, (95% CI)	*p*-Value
Total	15.73 (14.47, 16.99)	
Gender		0.3294
Male	16.19 (14.62, 17.76)	
Female	15.26 (13.69, 16.83)	
Age (years)		<0.0001
60~79	14.96 (13.72, 16.20)	
≥80	23.4 (19.40, 27.40)	
BMI		<0.0001
Underweight	6.81 (4.46, 9.16)	
Normal	11.04 (9.72, 12.36)	
Overweight	19.39 (17.67, 21.11)	
Obese	24.78 (21.61, 27.95)	
Education		0.0033
Primary school or below	14.8 (13.37, 16.24)	
Middle school	17.11 (14.87, 19.35)	
High school or higher	18.93 (16.42, 21.43)	
Income (CNY)		<0.0001
Low	12.12 (10.66, 13.59)	
Medium	16.07 (14.41, 17.72)	
High	21.02 (18.67, 23.37)	
Marital status		0.6513
Living with spouse	15.65 (14.38, 16.91)	
Other status	16.29 (13.38, 19.20)	
Current smoker		0.27
No	16.02 (14.69, 17.36)	
Yes	14.84 (12.81, 16.87)	
Alcohol drinking		0.0019
No	14.79 (13.37, 16.13)	
Yes	17.85 (15.93, 19.78)	
Physical activity		0.0743
Low	16.12 (14.09, 18.16)	
Moderate	17.02 (15.04, 19.01)	
High	14.72 (13.35, 16.09)	
Sedentary behavior (h)		0.0004
<2	11.34 (9.32, 13.36)	
2~3	15.55 (13.81, 17.29)	
≥4	17 (15.25, 18.76)	
Sleeping time (h)		0.3426
<6	15.33 (12.73, 17.93)	
6~9	15.42 (14.08, 16.77)	
≥10	17.59 (14.19, 20.99)	
NCDs		<0.0001
Less than one disease	11.24 (10.12, 12.37)	
Over two diseases	21.52 (19.58, 23.45)	

**Table 3:** Association between different dietary patterns and hyperuricemia by logistic regression.

Dietary Pattern	Group of Quartile	No. of Cases	Model 1	Model 2	Model 3
			OR (95% CI)	OR (95% CI)	OR (95% CI)
Typical Chinese	Q1	899	reference	reference	reference
	Q2	982	1.13 (1.02, 1.25)	1.06 (0.96, 1.18)	1.00 (0.90, 1.12)
	Q3	742	0.8 (0.72, 0.89)	0.66 (0.59, 0.74)	0.60 (0.53, 0.68)
	Q4	415	0.41 (0.37, 0.47)	0.34 (0.30, 0.38)	0.32 (0.28, 0.37)
	*p* for trend	-	<0.0001	<0.0001	<0.0001
Modern Chinese	Q1	706	reference	reference	reference
	Q2	784	1.14 (1.02, 1.28)	1.13 (1.01, 1.27)	1.10 (0.98, 1.23)
	Q3	798	1.17 (1.05, 1.31)	1.13 (1.01, 1.26)	1.04 (0.92, 1.17)
	Q4	750	1.08 (0.97, 1.21)	0.96 (0.86, 1.08)	0.81 (0.71, 0.93)
	*p* for trend	-	0.1462	0.4453	0.0021
Western	Q1	726	reference	reference	reference
	Q2	733	1.02 (0.91, 1.14)	1.03 (0.92, 1.16)	1.05 (0.93, 1.18)
	Q3	734	1.02 (0.92, 1.14)	0.99 (0.88, 1.10)	0.97 (0.86, 1.09)
	Q4	845	1.21 (1.09, 1.35)	1.11 (1.00, 1.24)	1.04 (0.93, 1.17)
	*p* for trend	-	0.0009	0.1207	0.8218
Animal products and alcohol	Q1	651	reference	reference	reference
	Q2	733	1.16 (1.04, 1.30)	1.19 (1.06, 1.33)	1.18 (1.05, 1.32)
	Q3	773	1.24 (1.10, 1.38)	1.27 (1.13, 1.42)	1.25 (1.11, 1.41)
	Q4	881	1.45 (1.30, 1.62)	1.49 (1.33, 1.68)	1.49 (1.31, 1.70)
	*p* for trend	-	<0.0001	<0.0001	<0.0001
Tuber and fermented vegetables	Q1	899	reference	reference	reference
	Q2	769	0.84 (0.75, 0.93)	0.87 (0.78, 0.97)	0.91 (0.82, 1.02)
	Q3	735	0.79 (0.71, 0.88)	0.85 (0.76, 0.95)	0.89 (0.80, 1.00)
	Q4	635	0.67 (0.60, 0.75)	0.73 (0.65, 0.82)	0.78 (0.69, 0.88)
	*p* for trend	-	<0.0001	<0.0001	<0.0001

Model 1: unadjusted model; Model 2: adjusted for age, gender, and BMI; Model 3: further adjusted for urban and rural, income, education, marital status, smoke, alcohol-drinking, static status, sleeping time, and total energy intake groups.

## Data Availability

The data are not allowed to be disclosed according to the National Institute for Nutrition and Health, Chinese Center for Disease Control and Prevention.

## Supplementary Materials

The following supporting information can be downloaded at: https://www.mdpi.com/article/10.3390/nu14040844/s1, Table S1: Distribution of weighted prevalence of hyperuricemia in Chinese mainland; Table S2: Factor loading of food item in each dietary pattern; Table S3: Distribution of dietary patterns among Chinese mainland; Table S4: Proportion of participants in each dietary pattern who reached the RNI/AI standard of nutrients.
