# Supplementary material for: Dietary Pattern Associated with the Risk of Hyperuricemia in Chinese Elderly: Result from China Nutrition and Health Surveillance 2015–2017
Source: Nutrients. 2022 Feb 17;14(4):844. doi: 10.3390/nu14040844 (PMC8875556; doi:10.3390/nu14040844)
Supplement: Supplementary file 1 [file nutrients-14-00844-s001.zip › nutrients-1579839-supplementary.pdf]

Table S1. Distribution of weighted prevalence of hyperuricemia in Chinese mainland

Province	Prevalence %, 95% CI
Beijing	21.32 (13.09, 29.54)
Tianjin	11.51 (6.60, 16.42)
Hebei	5.97 (2.65, 9.30)
Shanxi	6.12 (3.78, 8.46)
Inner Mongolia	12.44 (8.69, 16.19)
Liaoning	15.29 (10.95, 19.62)
Jilin	14.56 (8.31, 20.81)
Heilongjiang	14.29 (10.94, 17.63)
Shanghai	19.85 (18.02, 21.67)
Jiangsu	22.06 (18.35, 25.76)
Zhejiang	23.74 (17.36, 30.11)
Anhui	15.18 (12.02, 18.35)
Fujian	32.67 (27.31, 38.04)
Jiangxi	21.16 (14.44, 27.88)
Shandong	7.8 (3.79, 11.82)
Henan	9.27 (5.34, 13.21)
Hubei	13.19 (8.00, 18.37)
Hunan	19.38 (15.36, 23.40)
Guangdong	24.57 (14.48, 34.66)
Guangxi	22.21 (17.54, 26.89)
Hainan	30.71 (25.20, 36.22)
Chongqing	16.82 (10.42, 23.23)
Sichuan	14.64 (11.76, 17.52)
Guizhou	18.75 (8.71, 28.78)
Yunnan	23.96 (19.87, 28.05)
Tibet	17.91 (10.69, 25.14)
Shaanxi	8.42 (1.11, 15.73)
Gansu	5.52 (3.29, 7.75)
Qinghai	31.89 (18.10, 45.67)
Ningxia	9.51 (4.41, 14.60)
Xinjiang	12.33 (4.66, 20.00)
Hong Kong	N/A
Macao	N/A
Taiwan	N/A

Table S2. Factor loading of food item in each dietary pattern

Food item	Dietary pattern				
	Typical Chinese	Modern Chinese	Western	Animal products & alcohol	Tuber & fermented vegetables
Rice	-0.74				
Wheat	0.71				
Coarse grain	0.47	0.31			
Mixed beans		0.56			0.20
Tuber	0.24				0.49
Fried staple	0.39			0.34	
Legume and product		0.59			
Fresh vegetable	-0.23	0.38			
Dry vegetable					0.25
Fermented vegetable					0.55
Bacteria		0.61			
Fresh fruit		0.36	0.41		
Dry fruit		0.33	0.21		
Dairy and product		0.24	0.37		-0.23
Red meat	-0.21			0.53	
Poultry				0.48	-0.21
Processed meat			0.27	0.25	
Organ				0.58	
Aquatic product	-0.26	0.31		0.26	-0.31
Fresh egg	0.28	0.24	0.22		
Processed egg				0.27	
Western staple			0.54		
Nuts and seeds		0.21	0.28		
Snack			0.50		0.20
Soft beverage			0.48		
Alcohol			-0.20	0.50	
Animal fat	-0.33				0.43

Food item with a factor loading of more than 0.2 in each dietary pattern was displayed in the table

Table S3. Distribution of dietary patterns among Chinese mainland (N, %)

Province	Dietary pattern				
	Typical Chinese	Modern Chinese	Western	Animal products & alcohol	Tuber & fermented vegetables
Beijing	216 (35.64%)	133 (21.95%)	161 (26.57%)	81 (13.37%)	15 (2.48%)
Tianjin	261 (43.07%)	163 (26.9%)	96 (15.84%)	79 (13.04%)	7 (1.16%)
Hebei	450 (62.5%)	101 (14.03%)	67 (9.31%)	43 (5.97%)	59 (8.19%)
Shanxi	331 (64.4%)	85 (16.54%)	38 (7.39%)	6 (1.17%)	54 (10.51%)
Inner Mongolia	174 (38.24%)	23 (5.05%)	65 (14.29%)	45 (9.89%)	148 (32.53%)
Liaoning	144 (18.92%)	142 (18.66%)	201 (26.41%)	85 (11.17%)	189 (24.84%)
Jilin	96 (17.36%)	88 (15.91%)	112 (20.25%)	54 (9.76%)	203 (36.71%)
Heilongjiang	268 (38.56%)	52 (7.48%)	129 (18.56%)	60 (8.63%)	186 (26.76%)
Shanghai	36 (4.74%)	275 (36.23%)	308 (40.58%)	106 (13.97%)	34 (4.48%)
Jiangsu	225 (18.58%)	421 (34.76%)	200 (16.52%)	224 (18.5%)	141 (11.64%)
Zhejiang	76 (9.1%)	196 (23.47%)	147 (17.6%)	207 (24.79%)	209 (25.03%)
Anhui	224 (21.85%)	182 (17.76%)	119 (11.61%)	212 (20.68%)	288 (28.1%)
Fujian	20 (3.41%)	138 (23.55%)	111 (18.94%)	187 (31.91%)	130 (22.18%)
Jiangxi	57 (8.68%)	81 (12.33%)	111 (16.89%)	140 (21.31%)	268 (40.79%)
Shandong	470 (72.87%)	77 (11.94%)	35 (5.43%)	58 (8.99%)	5 (0.78%)
Henan	705 (74.52%)	123 (13%)	61 (6.45%)	39 (4.12%)	18 (1.9%)
Hubei	118 (20.56%)	135 (23.52%)	76 (13.24%)	115 (20.03%)	130 (22.65%)
Hunan	85 (9.15%)	90 (9.69%)	113 (12.16%)	249 (26.8%)	392 (42.2%)
Guangdong	33 (5.83%)	53 (9.36%)	123 (21.73%)	286 (50.53%)	71 (12.54%)
Guangxi	23 (4.14%)	57 (10.27%)	53 (9.55%)	297 (53.51%)	125 (22.52%)
Hainan	9 (3.23%)	33 (11.83%)	29 (10.39%)	160 (57.35%)	48 (17.2%)
Chongqing	37 (5.81%)	61 (9.58%)	79 (12.4%)	105 (16.48%)	355 (55.73%)
Sichuan	88 (9.25%)	82 (8.62%)	164 (17.25%)	201 (21.14%)	416 (43.74%)
Guizhou	12 (3.81%)	8 (2.54%)	31 (9.84%)	74 (23.49%)	190 (60.32%)
Yunnan	13 (2.44%)	28 (5.25%)	93 (17.45%)	75 (14.07%)	324 (60.79%)
Tibet	37 (38.14%)	1 (1.03%)	17 (17.53%)	30 (30.93%)	12 (12.37%)
Shaanxi	343 (69.43%)	73 (14.78%)	23 (4.66%)	5 (1.01%)	50 (10.12%)
Gansu	182 (79.13%)	8 (3.48%)	11 (4.78%)	4 (1.74%)	25 (10.87%)
Qinghai	201 (64.22%)	15 (4.79%)	56 (17.89%)	21 (6.71%)	20 (6.39%)
Ningxia	188 (54.81%)	13 (3.79%)	47 (13.7%)	19 (5.54%)	76 (22.16%)
Xinjiang	150 (49.83%)	48 (15.95%)	44 (14.62%)	40 (13.29%)	19 (6.31%)
Hong Kong	N/A				
Macao	N/A				
Taiwan	N/A				

Table S4. Proportion of participants in each dietary pattern who reached the RNI/AI standard of nutrients (N, %)

Subject	Dietary Pattern					*P*-value
	Typical Chinese	Modern Chinese	Western	Animal products & alcohol	Tuber & fermented vegetables	
**N (%)**	5272 (28.21%)	2985 (15.97%)	2920 (15.62%)	3307 (17.69%)	4207 (22.51%)	-
**Calcium**						**<0.0001**
Not reached	5204 (98.71%)	2452 (82.14%)	2556 (87.53%)	3213 (97.16%)	4143 (98.48%)	
Reached	68 (1.29%)	533 (17.86%)	364 (12.47%)	94 (2.84%)	64 (1.52%)	
**Magnesium**						**<0.0001**
Not reached	3191 (60.53%)	653 (21.88%)	1497 (51.27%)	1922 (58.12%)	2705 (64.3%)	
Reached	2081 (39.47%)	2332 (78.12%)	1423 (48.73%)	1385 (41.88%)	1502 (35.7%)	
**Zinc**						**<0.0001**
Not reached	4523 (85.79%)	952 (31.89%)	1517 (51.95%)	1769 (53.49%)	2682 (63.75%)	
Reached	749 (14.21%)	2033 (68.11%)	1403 (48.05%)	1538 (46.51%)	1525 (36.25%)	
**Selenium**						**<0.0001**
Not reached	4835 (91.71%)	2253 (75.48%)	2497 (85.51%)	2653 (80.22%)	4127 (98.1%)	
Reached	437 (8.29%)	732 (24.52%)	423 (14.49%)	654 (19.78%)	80 (1.9%)	
**Vitamin B_1**						**<0.0001**
Not reached	4008 (76.02%)	1148 (38.46%)	2020 (69.18%)	1900 (57.45%)	3053 (72.57%)	
Reached	1264 (23.98%)	1837 (61.54%)	900 (30.82%)	1407 (42.55%)	1154 (27.43%)	
**Vitamin C**						**<0.0001**
Not reached	4367 (82.83%)	1189 (39.83%)	1584 (54.25%)	2384 (72.09%)	3049 (72.47%)	
Reached	905 (17.17%)	1796 (60.17%)	1336 (45.75%)	923 (27.91%)	1158 (27.53%)	
**Vitamin E**						**<0.0001**
Not reached	448 (8.5%)	46 (1.54%)	178 (6.1%)	325 (9.83%)	725 (17.23%)	
Reached	4824 (91.5%)	2939 (98.46%)	2742 (93.9%)	2982 (90.17%)	3482 (82.77%)	
**Dietary fiber**						**<0.0001**
Not reached	5160 (97.88%)	2153 (72.13%)	2706 (92.67%)	3222 (97.43%)	4111 (97.72%)	
Reached	112 (2.12%)	832 (27.87%)	214 (7.33%)	85 (2.57%)	96 (2.28%)	

For Calcium, Magnesium, Zinc, Selenium, Vitamin B1, Vitamin C and dietary fiber were calculated based on Recommended Nutrient Intake (RNI), For Vitamin E was calculated based on Adequate Intake (AI).
